# Cnicin: a promising drug for promoting nerve repair

**DOI:** 10.3389/fcell.2025.1558525

**Published:** 2025-04-17

**Authors:** Dietmar Fischer

**Affiliations:** Center of Pharmacology, Institute for Pharmacology 2, Medical Faculty and University of Cologne, Cologne, Germany

**Keywords:** cnicin, axon regeneration, microtubule detyrosination, vasohibins, parthenolide, GSK3-glycogen synthase kinase 3

## Abstract

Traumatic peripheral nerve injuries frequently result in irreversible functional deficits. While neurons possess an intrinsic capacity for axonal regeneration, the temporal constraints and the slow pace of neurite outgrowth often impede the complete restoration of sensory and motor capabilities. This impairment, often culminating in chronic disability, represents a significant clinical challenge, as there are currently no approved pharmacological interventions available to accelerate axon regeneration and improve functional recovery. This perspective focuses on recent scientific advancements that have identified sesquiterpene lactones, a family of naturally derived plant metabolites, as potential therapeutic candidates for treating peripheral nerve trauma. Preclinical investigations employing parthenolide and cnicin have revealed that these compounds can substantially augment axonal extension and functional recovery in diverse *in vivo* animal paradigms and primary human neuronal cultures. The favorable bioavailability of cnicin following oral administration, coupled with its notable tolerability at dosages considerably largely surpassing the therapeutic range, underscores its substantial potential as an effective pharmacological treatment for addressing the challenges associated with nerve regeneration and restoring sensory and motor functions.

## Insufficient axonal growth rate limits functional recovery

Traumatic axonal insults to nerves disrupt neuronal connectivity with target tissues, resulting in motor and sensory deficits. While the peripheral nervous system possesses an intrinsic capacity for axonal regeneration, such injuries nonetheless often lead to permanent impairments, diminishing the quality of patients’ lives and imposing a substantial socioeconomic burden (Grinsell and Keating, 2014; Black and Lasek, 1979). For instance, peripheral nerve injuries in the upper extremities are relatively common, particularly in the context of occupational accidents, and can result in significant morbidity and long-term expenses (Tapp et al., 2019). One study revealed that 30% of patients with work-related nerve injuries experienced persistent disabilities. The projected lifetime cost per patient with severe injury, encompassing treatment, rehabilitation, and compensation, is estimated at approximately € 102,167, underscoring the considerable economic burden of these injuries (Bergmeister et al., 2020). Despite their principal capacity for axon regeneration, the recovery rate is influenced by patient’s age and the characteristics and severity of the injury (Grinsell and Keating, 2014). Moreover, the likelihood of functional restoration decreases as the distance between the lesion site and the target organ increases, owing to the slow velocity of axonal elongation, typically not exceeding 2–3 mm per day. Consequently, reinnervation of the target tissue following traumatic injury to the extremities may require a period of several weeks to months. This protracted timeframe, however, presents a significant challenge, as the necessary regenerative support provided by Schwann cells attenuates over time. Thus, axons do not reach their targets, ultimately leading to muscle atrophy in denervated regions (Sulaiman and Gordon, 2013). Furthermore, the temporal decline in Schwann cell support delays functional recovery and can also prevent axons from reaching their appropriate targets, potentially culminating in permanent functional loss. Moreover, regenerating axons often exhibit aberrant growth patterns, forming neuromas near the lesion site and contributing to chronic, refractory pain (Wei et al., 2024). Consequently, the restricted axonal growth velocity can substantially impair patients’ quality of life and is associated with significant socioeconomic burdens and extended periods of occupational disability (Bergmeister et al., 2020).

## Still existing need of applicable drugs speeding up axonal regeneration

Despite extensive research endeavors over the past decades, therapeutic strategies to accelerate axonal elongation and enhance functional recovery have demonstrated limited clinical efficacy or have been deemed unsuitable due to adverse side effects. For instance, numerous preclinical studies have investigated neurotrophic factors, such as nerve growth factor and brain-derived neurotrophic factor, to promote axonal regeneration (Zheng et al., 2016). These factors exert essential functions in neuronal survival and development, and their exogenous administration has yielded beneficial effects on nerve repair in preclinical models (Faroni et al., 2015). However, the clinical application of neurotrophic factors in humans is hampered by significant obstacles, including a short half-life, inefficient delivery modalities, and potential adverse effects (Sarhane et al., 2023). Another promising pharmacological approach for promoting axonal regeneration following nerve injury involves the administration of tacrolimus. This immunosuppressant medication has demonstrated neuroregenerative properties in preclinical investigations and clinical case reports. While the mechanisms underlying its neuroregenerative effects remain incompletely elucidated, these positive effects on regenerating nerve fibers are distinct from its immunosuppressive actions and primarily affect the injured neuron (Daeschler et al., 2023; Gold et al., 1994). Nevertheless, systemic administration of tacrolimus is restricted by significant adverse effects, such as nephrotoxicity and immunosuppression. Therefore, a critical unmet clinical need for agents that promote axonal regeneration with an acceptable safety profile remains.

## Inhibition of microtubule detyrosination accelerates axon regeneration and recovery

The discovery that constitutively active glycogen synthase kinase 3 markedly accelerates axonal regeneration in peripheral nerves, coupled with understanding the underlying mechanism, has unveiled novel avenues for pharmacological interventions (Diekmann and Fischer, 2015). GSK3 is a serine/threonine kinase comprising two isoforms encoded by distinct genes, GSK3α and GSK3β. Investigations utilizing knockin mice expressing constitutively active variants of GSK3 (GSK3^S/A^) have revealed a marked acceleration of axonal regeneration in the injured sciatic nerve and enhanced functional recovery in adult mice (Gobrecht et al., 2014). This effect on axonal elongation stems from GSK3’s influence on microtubule dynamics within the growing axons. The underlying mechanism involves the kinase’s capacity to phosphorylate microtubule-associated protein 1B (MAP1B), which subsequently diminishes the level of microtubule detyrosination - a post-translational modification known to attenuate microtubule dynamics in axonal growth cones, thereby limiting the rate of axonal extension (Gobrecht et al., 2016; Scales et al., 2009; Trivedi et al., 2005). Microtubule detyrosination in neurons is regulated by distinct enzymatic activities that either add or remove the C-terminal tyrosine residue of tubulin. Tubulin tyrosine ligase catalyzes the addition of a tyrosine residue to the detyrosinated α-subunit of tubulin dimers before their incorporation into microtubule plus-ends, thereby restoring the tyrosinated state at the C-terminal extremity (Utreras et al., 2008; Gonzalez-Billault et al., 2004). Although there is no direct evidence of an interaction between phosphorylated MAP1B (pMAP1B) and tubulin tyrosine ligase, pMAP1B reduces microtubule detyrosination by associating with tyrosinated microtubules and impeding the accessibility of detyrosinating enzymes (Barnat et al., 2016). These tyrosine-removing enzymes, also termed carboxypeptidases, have been identified as vasohibin-1 (VASH 1) and vasohibin-2 (VASH 2) (Aillaud et al., 2017). The small vasohibin-binding protein (SVBP) is an essential cofactor for vasohibin enzymatic activity, suggesting a tightly regulated enzymatic collaboration that ensures precise control over microtubule dynamics within neurons (Li et al., 2019). A partial reduction in vasohibin enzyme (VASH1 or VASH2) or SVBP expression subtly alters the equilibrium between detyrosinated and tyrosinated microtubules, mirroring the state observed in regenerating axonal growth cones of postnatal neurons (Gobrecht et al., 2024a), which exhibit elevated growth rates compared to mature neurons. Correspondingly, the axonal growth rate of adult neurons was also augmented to levels comparable to those observed in the postnatal mice (Gobrecht et al., 2024a). However, complete knockdown of either enzyme shifts the balance of detyrosinated and tyrosinated microtubules to suboptimal levels, resulting in either no discernible benefit or even detrimental effects on axonal growth, highlighting the delicate balance required for optimal microtubule dynamics and axonal elongation (Gobrecht et al., 2024a).

## Sesquiterpene lactones as promising drugs for promoting nerve repair

The observation that partial vasohibin knockdown enhances axonal regeneration prompted investigations into whether the pharmacological intervention could replicate these effects. The sesquiterpene lactone parthenolide, a naturally occurring compound derived from the plant *Tanacetum parthenium*, was identified as a vasohibin inhibitor, influencing the detyrosination-tyrosination equilibrium of microtubules (Li et al., 2019; Fonrose et al., 2007). Different studies have demonstrated that parthenolide promotes axonal growth in cultured dorsal root ganglion neurons from adult rats and mice and even in primary retinal ganglion cells derived from rodents and humans (Gobrecht et al., 2016; Gobrecht et al., 2024a; Leibinger et al., 2023). Furthermore, *in vivo* experiments in various rodent models of peripheral nerve injury have also demonstrated the considerable therapeutic potential of parthenolide. Daily intravenous injections of 2 μg/kg markedly accelerated motor and sensory function recovery following sciatic nerve injury (Gobrecht et al., 2024a). Parthenolide retained its efficacy even when treatment was initiated several days after nerve injury. Despite its promising preclinical findings in accelerating axonal regeneration, parthenolide faces significant limitations due to its low oral bioavailability, restricting its administration to parenteral routes. Given that repeated daily administration yielded the most robust outcomes, the necessity of repeated intravenous applications is problematic regarding costs and treatment adherence limitations. Another sesquiterpene lactone, cnicin, a naturally occurring compound found in blessed thistle (*Cnicus benedictus*), enhances axonal regeneration in diverse species, likely also via inhibition of VASHs (Gobrecht et al., 2024b). Compared to parthenolide, cnicin presents two key advantages as a potential therapeutic agent for nerve regeneration. First, cnicin exhibits an oral bioavailability of 84.7% in rats. Second, it lacks the potentially mutagenic and allergenic epoxy group in parthenolide, rendering it a potentially safer alternative and a strong candidate for further drug development (Gobrecht et al., 2024b). Cnicin promoted axonal growth in rodent neuronal cultures and, similar to parthenolide, in cultured adult primary human neurons (Gobrecht et al., 2024b). Moreover, it demonstrated efficacy in rats, mice, and rabbits at 2 μg/kg daily doses. Intravenous administration of cnicin at 4 mg/kg (2000-fold higher than the effective oral dose) for 2 weeks did not induce observable toxicity or alterations in rat body weight, suggesting a favorable safety profile for human application. Therefore, while preclinical investigations have demonstrated the substantial potential of parthenolide in promoting axonal regeneration, its clinical translation is hindered by its limited oral bioavailability. Conversely, cnicin, with its advantageous pharmacokinetic properties, high oral bioavailability, absence of a potentially mutagenic epoxy group, and demonstrated efficacy in enhancing axonal regeneration across multiple species, emerges as a more promising candidate for further development. However, rigorous clinical trials are essential to confirm its safety and efficacy in humans, establish optimal dosing regimens, and evaluate its therapeutic potential across various nerve injury types and clinical contexts. Identifying sesquiterpene lactones as a promising therapeutic option represents a significant step towards developing effective treatments for nerve damage, offering hope for improved patient outcomes and enhanced quality of life.

## Data Availability

The original contributions presented in the study are included in the article/supplementary material, further inquiries can be directed to the corresponding author.
